# CYP105—diverse structures, functions and roles in an intriguing family of enzymes in *Streptomyces*

**DOI:** 10.1111/jam.12662

**Published:** 2014-11-04

**Authors:** Suzy C Moody, E Joel Loveridge

**Affiliations:** 1 Department of Biosciences, College of Science, Swansea UniversitySwansea, UK; 2 School of Chemistry, Cardiff UniversityCardiff, UK

**Keywords:** antimicrobial, bioremediation, biotransformation, P450, polyketide synthesis, secondary metabolism, *Streptomyces*, xenobiotic degradation

## Abstract

The cytochromes P450 (CYP or P450) are a large superfamily of haem-containing enzymes found in all domains of life. They catalyse a variety of complex reactions, predominantly mixed-function oxidations, often displaying highly regio- and/or stereospecific chemistry. In streptomycetes, they are predominantly associated with secondary metabolite biosynthetic pathways or with xenobiotic catabolism. Homologues of one family, CYP105, have been found in all *Streptomyces* species thus far sequenced. This review looks at the diverse biological functions of CYP105s and the biosynthetic/catabolic pathways they are associated with. Examples are presented showing a range of biotransformative abilities and different contexts. As biocatalysts capable of some remarkable chemistry, CYP105s have great biotechnological potential and merit detailed study. Recent developments in biotechnological applications which utilize CYP105s are described, alongside a brief overview of the benefits and drawbacks of using P450s in commercial applications. The role of CYP105s *in vivo* is in many cases undefined and provides a rich source for further investigation into the functions these enzymes fulfil and the metabolic pathways they participate in, in the natural environment.

## Introduction

The cytochromes P450 are a superfamily of haemoproteins, first identified in mammalian liver microsomes (Omura and Sato 1962). They were found to be involved in the production of steroid hormones such as progesterone and play important roles in drug and xenobiotic metabolism (Omura 1993). P450s have since been found in animals, plants, fungi and bacteria, displaying a diverse range of functions from biosynthesis of sterols to the breakdown of aromatic petrochemicals (Ichinose 2012). They catalyse a variety of NADPH/NADH- and O_2_-dependent reactions including hydroxylation; alcohol and carbonyl oxidation; epoxidation; dealkylation; heteroatom oxidation; desaturation; ring cleavage, expansion, coupling and formation; dehydration; and even reduction (Guengerich and Munro 2013). According to the widely accepted P450 nomenclature system (Nelson 2009), a pairwise amino acid sequence identity of 40% is required to assign proteins within the same P450 family (e.g. CYP105), and 55% identity is required for assignment within the same subfamily (e.g. CYP105A).

**Figure 1:**
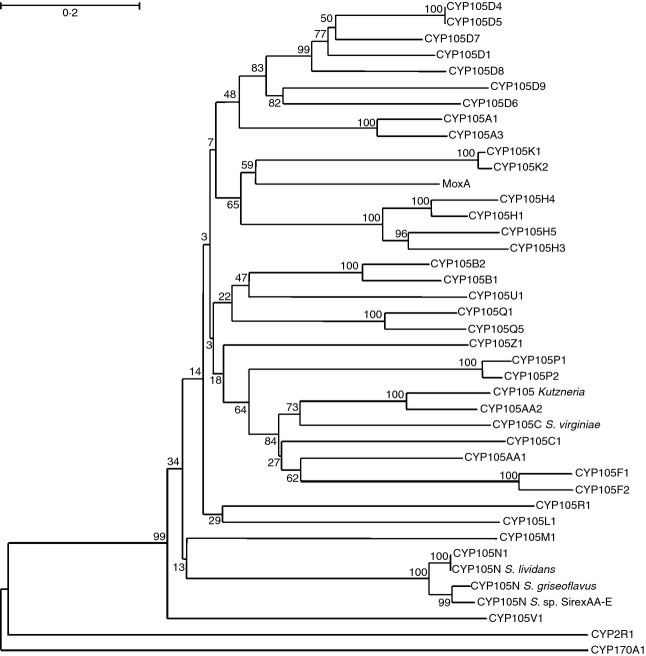
Phylogenetic tree of CYP105 homologues in *Streptomyces*. Amino acid sequences for CYP105s in *Streptomyces spp*. were aligned (Sievers *et al*. 2013) and a phylogenetic tree generated. The tree was obtained in Mega6 using the neighbour-joining method (Tamura *et al*. 2013). The values shown are confidence percentages based on a bootstrap value of 500 repetitions. The scale bar (top left) indicates a genetic change of 0·2. Only CYP105s assigned to a subfamily by Nelson (http://drnelson.uthsc.edu/CytochromeP450.html) are included, so the list should not be considered exhaustive. Also included were examples of CYP105 enzymes from other actinomycete genera, MoxA from *Nonomuraea recticatena* and a CYP105 from *Kutzneria*. The final proteins included as comparators were CYP170A1, a well-characterized P450 from *Streptomyces coelicolor*, and CYP2R1, a human P450 with low level amino acid homology to CYP105 enzymes.

In bacteria, the occurrence of P450s is highly variable. While the *Escherichia coli* genome has no identified P450s, many other species contain multiple P450 homologues. Some of the first examples of bacterial P450s to be identified include P450cam (CYP101A1) in *Pseudomonas putida* (Sligar *et al*. 1974) and P450BM3 (CYP102A1) in *Bacillus megaterium* (Fulco 1991). Every actinomycete species that has had its genome sequenced has revealed an abundance of P450s. For example, *Streptomyces coelicolor* has 18 P450s (Lamb *et al*. 2002), *Mycobacterium tuberculosis* has 20 (McLean *et al*. 2006), and *Streptomyces avermitilis* has 33 putative P450s (Ikeda *et al*. 2003; Lamb *et al*. 2003). CYP105s are predominantly found in bacteria belonging to the phylum Actinobacteria and the order Actinomycetales, with the exception of CYP105T1 which is found in *Burkholderia fungorum*, in the phylum Proteobacteria (based on those proteins named in the P450 nomenclature system at http://drnelson.uthsc.edu/bacterial.P450s.2011.htm). A review of current literature suggests that CYP105 may be uniquely conserved as the only P450 family with representatives in every streptomycete species thus far investigated (also in Takamatsu *et al*. 2011). The CYP105 family has at least 17 subfamilies represented in streptomycetes (Fig.1 and Table1), with their roles falling broadly into two categories: biotransformation or degradation of xenobiotics, and biosynthesis of specialized bioactive molecules. This functional diversity suggests there is considerable variation in structure within the family.

**Table 1:** The complete CYP105 proteins from *Streptomyces* listed on the Cytochrome P450 homepage (http://drnelson.uthsc.edu/CytochromeP450.html), with those mentioned in the text that have yet to be assigned within official P450 nomenclature included

CYP105	Uniprot ID	Species of *Streptomyces*	Function (if known)
A1	P18326	*griseolus*	Wide range (see text)*,†
A3	Q59831	*carbophilus*	Pravastatin synthesis†
B1	P18327	*griseolus*	Sulfonylurea oxidation*
B2	Q595R4	*tubercidicus*	Avermectin oxidation†
C1	P23296	sp.	
C-	A6YRR3	*virginiae*	Steroid oxidation*
D1	P26911	*griseus*	Xenobiotic degradation*
D4	O85697	*lividans*	
D5	Q9EWS4	*coelicolor*	Fatty acids‡
D6	Q79ZT5	*avermitilis*	Filipin C1′‡
D7	Q825I8	*avermitilis*	Pentalenic acid, xenobiotics*^,^‡
D8	Q595S4	*tubercidicus* strain I-1529	Avermectin oxidation†
D9	Q8KSY0	sp. JP95	Griseorhodin‡
F1	Q9X5P8	*lavendulae*	Unknown polyketide
F2	Q70AS0	*peucetius*	Oleandomycin oxidation‡
H1	Q9L4W8	*noursei* ATCC 11455	Nystatin‡
H3	Q9EW94	*natalensis*	Pimaricin‡
H4	Q93NX1	*nodosus*	Amphotericin‡
H5	Q9EWC4	*griseus*	Candidicin‡
K1	Q9X9P7	*tendae* strain Tue901	Nikkomycin‡
K2	Q8RNW8	*ansochromogenes*	Nikkomycin‡
L1	Q9ZHQ1	*fradiae*	Tylosin‡
M1	Q9KJ93	*clavuligerus*	Clavulanic acid‡
N1	Q9EWP1	*coelicolor*	Coelibactin‡
N-	D6ESR2	*lividans*	Putatively coelibactin‡
N-	D9XRK0	*griseoflavus*	Putatively coelibactin‡
N-	G2NQM5	SirexAA-E	Putatively coelibactin‡
P1	Q79ZT4	*avermitilis*	Filipin C26‡
P2	Q70AS3	*peucetius*	Flavone oxidation*
Q1	Q82MP9	*avermitilis*	
Q5	C9Z4Z8	*scabies*	
R1	Q93HF0	*avermitilis*	
U1	Q84G11	*hygroscopicus* strain NRRL3602	Geldanamycin‡
V1	Q6V1N2	sp. HK803	Phoslactomycin‡
Z1	C9ZGE5	*scabies* strain 87·22	
AA1	Q595R1	*tubercidicus* strain R922	Avermectin oxidation†
AA2	Q595S0	*tubercidicus* strain I-1529	Avermectin oxidation†

In the final column, *indicates a xenobiotic breakdown function, †a biotechnological use and ‡indicates the biosynthetic pathway the protein is associated with.

CYP105s are associated with a wide variety of pathways and processes, from steroid biotransformation to production of macrolide metabolites (see below). Individual P450s, including CYP105s, may also be capable of catalysing diverse reactions, with one enzyme capable of catalysing a range of reactions with a variety of structurally different compounds (e.g. Kittendorf and Sherman 2009). For example, CYP105A1 catalyses two sequential hydroxylations of vitamin D3 with differing specificity (Sugimoto *et al*. 2008), and CYP105D1 has been shown to be capable of both oxidation (Taylor *et al*. 1999) and dealkylation reactions (Ueno *et al*. 2005). The low level of substrate stringency exhibited by some of these enzymes makes them potentially interesting for engineering novel compounds from related substrates. Many other CYP105s, on the other hand, demonstrate high substrate and site specificity. This is often true of P450s which act as part of a biosynthetic process (Podust and Sherman 2012). For example, CYP105D6 and CYP105P1 in the filipin biosynthetic pathway perform highly regio- and stereospecific hydroxylations (Xu *et al*. 2010). This review aims to highlight the diversity of CYP105 structures and catalytic functions; CYP105s involved in xenobiotic degradation and their potential use in bioremediation; those involved in biosynthesis of useful bioactive molecules; and those which have proved amenable to biotechnological applications. While CYP105s have been known for many years, the last decade has seen a rapid rise in both structural and functional studies, with the result that several of these enzymes are now being utilized (and modified for use) in novel biotechnological applications.

## CYP105s involved in xenobiotic catabolism

**Figure 2:**
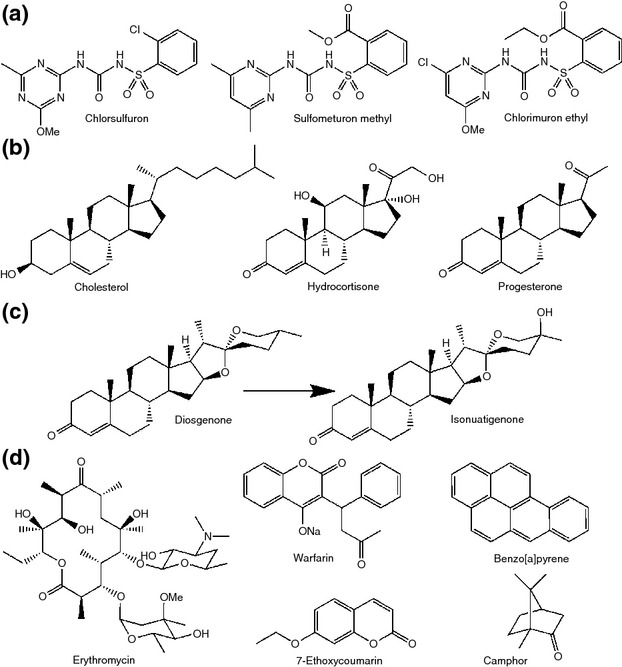
Selected xenobiotic substrates of CYP105s. (a) Sulfonylurea herbicide substrates of CYP105A1 from *Streptomyces griseolus*, (b) steroid substrates of CYP105C from *Streptomyces virginiae* IBL14, (c) reaction scheme for conversion of diosgenone to isonuatigenone by CYP105C from *S. virginiae* IBL14 and (d) substrates of CYP105D1 from *S. griseus* discussed in the text. Except in the case of diosgenone, where the modification is indicated, and 7-ethoxycoumarin, which is dealkylated, the site of modification by the CYP105 has not been identified.

The P450 superfamily is well known for its roles in detoxification of xenobiotic compounds (Omura 1993), and it is therefore unsurprising that early work on CYP105s focused on this ability. Although the CYP105 family has several examples of involvement in degradation of xenobiotic compounds, the ecological context of this function is not always clear, as an enzyme capable of metabolizing numerous xenobiotics is often researched more in the context of biotechnology and less for the specialized metabolism required in the environmental niche the cell occupies. For example, CYP105D1 from *Streptomyces griseus* has been shown to be capable of oxidizing a diverse range of xenobiotics (Fig.2) including camphor (Sariaslani *et al*. 1990), benzo[a]pyrene, erythromycin and warfarin (Taylor *et al*. 1999) and can catalyse the oxidative dealkylation of 7-ethoxycoumarin (Ueno *et al*. 2005). These studies, however, were conducted using heterologously expressed protein, so the role, substrate and catalytic function of the P450 in the native streptomycete cell remain unknown. More interesting, perhaps, is a study of the correlation between the presence of toxic herbicides and the action of CYP105A1 and CYP105B1 in *Streptomyces griseolus*. Not only are these enzymes capable of metabolizing sulfonylurea herbicides (Fig.2) to less toxic compounds (O'Keefe *et al*. 1988), but production of P450s in the cell was induced by the addition of the herbicide to the growth media (Romesser and O'Keefe 1986). This suggests that the cell has the ability to recognize and respond to the toxic compound (or closely related compounds) in the environment and that the observed *in vitro* xenobiotic biotransformation may indeed occur in this species in nature.

More recent developments include an excellent example of using knowledge of the species' ecological niche to inform further investigation. *Streptomyces virginiae* IBL14 was originally isolated from waste sludge from steroid manufacture (Wang *et al*. 2007) and was found to degrade a range of steroidal compounds including progesterone, cholesterol and hydrocortisone (Wang *et al*. 2009a) (Fig.2). *Streptomyces virginiae* IBL14 contains five predicted CYP105 homologues, of 33 P450s in total, and several were linked to the xenobiotic degradative abilities of the organism (Li *et al*. 2013). One was found to be clustered with a cholesterol oxidase, suggesting a role in oxidative transformation of cholesterol and possibly its analogues, although this was not followed up experimentally (Li *et al*. 2013). A CYP105C was isolated and its degradative abilities confirmed *in vitro*. The CYP105C was shown to catalyse conversion of diosgenin (a platform compound used in the synthesis of a variety of steroidal products) to isonuatigenone (a rare natural steroidal sapogenin) *via* hydroxylation of the tertiary C25 (Wang *et al*. 2007, 2009b) (Fig.2). Natural and synthetic steroids are regarded as significant water pollutants due to their ability to cause endocrine disruption, reproductive toxicity and damaging effects on mammalian cell development (see Sun *et al*. 2013 for review). In the light of this, *S. virginiae* IBL14 or processes utilizing the purified P450s may be useful for bioremediation of steroidal compounds in wastewater. While xenobiotic metabolism has been investigated as a property of CYP105 enzymes for decades, studies such as this show there is still much to discover.

## CYP105s in the biosynthetic pathways of specialized metabolites

**Figure 3:**
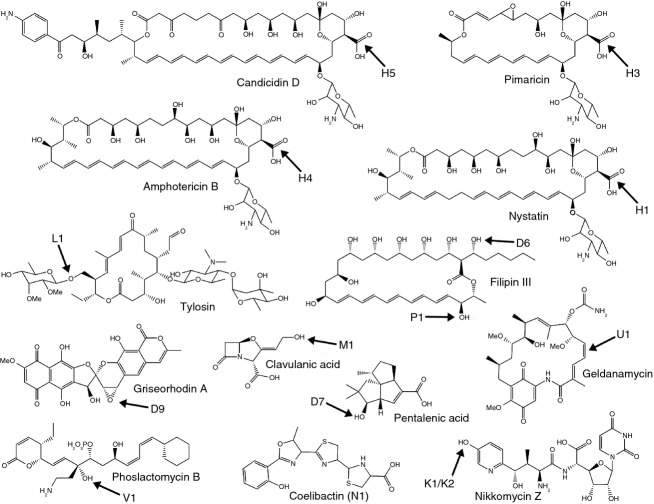
Secondary metabolites whose biosynthesis requires the action of a CYP105. The structural feature introduced by the CYP105 is arrowed, and the CYP105 involved is indicated. The site of modification of coelibactin is not known.

A driving factor in much of the research on streptomycetes has been the abundance of secondary metabolites produced by this genus. Over the last 10 years, there has been increasing appreciation of the intricate tailoring role of P450s in many biosynthetic pathways, as they are often associated with the final steps of specialized metabolite production, refining the final product by complex regio- and stereospecific oxidative tailoring (Zhao and Waterman 2007). There are now several examples of CYP105s known to play important roles in biosynthesis of bioactive molecules (Fig.3), and new pathways are still being revealed, suggesting further CYP105 involvement may be uncovered.

Characterized streptomycete CYP105s are predominantly involved in polyketide biosynthesis. Several members of the CYP105H subfamily catalyse oxidation of a methyl group to a carboxylic acid in the biosynthesis of a range of polyene antifungals (Fig.3) (Aparicio *et al*. 2000; Brautaset *et al*. 2000; Gil and Campelo 2002; Carmody *et al*. 2005). This three-step oxidation process has a strong precedent in the CYP51-catalysed oxidative demethylation of sterols (Akhtar *et al*. 1978; Lepesheva and Waterman 2007). Other members of the CYP105 family catalyse simpler hydroxylation reactions in streptomycete polyketide biosynthesis (Fig.3). Biosynthesis of the macrolide tylosin in *Streptomyces fradiae* involves hydroxylation of the C23 methyl group by CYP105L1 and subsequent glycosylation of the hydroxyl (Fouces *et al*. 1999). A further two members of the CYP105 family form part of the biosynthetic pathway of the polyene antifungal filipin in *Streptomyces avermitilis* (Xu *et al*. 2009). CYP105D6 and CYP105P1 in *S. avermitilis* catalyse regiospecific hydroxylations at C1′ and C26 of filipin, respectively (Xu *et al*. 2010).

Other known activities of CYP105s in polyketide biosynthesis (Fig.3) include an epoxidation reaction in griseorhodin biosynthesis, catalysed by CYP105D9 of *Streptomyces* sp. JP95 (Yunt *et al*. 2009), and an apparent hydroxylation–dehydration reaction catalysed by CYP105U1 during biosynthesis of geldanamycin by *Streptomyces hygroscopicus* (Li *et al*. 2012). In *Streptomyces* sp. HK803, CYP105V1 catalyses a hydroxylation reaction in the biosynthesis of phoslactomycin (Palaniappan *et al*. 2003). Other CYP105s are known to cluster with polyketide biosynthesis genes, but their products are unknown. For example, CYP105F1 of *Streptomyces lavendulae* is known to be part of a polyketide synthase (PKS) cluster adjacent to the cluster responsible for the biosynthesis of the antibiotic mitomycin (Mao *et al*. 1999), and CYP105R1 is part of the *pks*4 cluster in *S. avermitilis* (Lamb *et al*. 2003).

CYP105s are also involved in the biosynthesis of other classes of secondary metabolite (Fig.3). Members of the CYP105K subfamily catalyse an aromatic hydroxylation reaction in the biosynthesis of the peptidyl nucleoside antibiotic nikkomycin, and CYP105M1 catalyses an oxidative deamination in the biosynthesis of the clinically important *β*-lactamase inhibitor clavulanic acid by *Streptomyces clavuligerus*. CYP105D5 of *S. coelicolor* catalyses oxidation of various fatty acids *in vitro*, and the *in vitro* products were shown to also be produced *in vivo*, suggesting that this is the true physiological role of the enzyme (Chun *et al*. 2007). Another member of the CYP105D subfamily, CYP105D7 of *S. avermitilis*, is involved in the production of pentalenic acid (Takamatsu *et al*. 2011). This is a known cometabolite of pentalenolactone biosynthesis in other organisms and is formed by CYP105D7-catalysed hydroxylation at C1 of 1-deoxypentalenic acid, although the encoding gene is not part of the pentalenolactone biosynthesis cluster (Takamatsu *et al*. 2011). In addition to its biosynthetic role, CYP105D7 was also able to catalyse oxidation of a range of unrelated compounds, although it was unable to oxidize pentalenolactone-related molecules other than 1-deoxypentalenic acid (Takamatsu *et al*. 2011). Pentalenolactones are sesquiterpenoid antibiotics produced by numerous streptomycetes. Sesquiterpenoids may have an important and as yet undefined role in streptomycete biology as several terpenoid biosynthetic pathways have been shown to be highly conserved across many diverse species (e.g. geosmin, Gerber and Lechevalier 1965; albaflavenone, Moody *et al*. 2012). Terpenoids are often signalling molecules in nature with roles as attractants, repellents and for communicating information to a dispersed community (see Gershenzon and Dudareva 2007, for review). Playing a part in the biosynthesis of these potential signalling molecules would define a new and exciting role for P450 enzymes in streptomycete biology.

CYP105N1 in *S. coelicolor* is an interesting example of an enzyme involved in a cryptic biosynthetic pathway. The coelibactin biosynthetic gene cluster of which it is a part was identified by genome sequencing (Bentley *et al*. 2002). This nonribosomal peptide synthetase (NRPS) cluster is thought to produce a zinc-chelating siderophore, and gene expression of the pathway has indeed been shown to be responsive to zinc (Kallifidas *et al*. 2010), but the pathway remains cryptic as the end product has yet to be detected (Zhao *et al*. 2012). Bioinformatic analysis shows that this subfamily is well conserved, with three other species of streptomycetes possessing a homologue with over 90% identity to CYP105N1: *Streptomyces* sp. *Sirex* AA-E, *Streptomyces griseoflavus* and two strains of *Streptomyces lividans* (TK24, which is included here, and 1326) (Fig.1 and Table1). The extremely high sequence homology within the CYP105N subfamily suggests that they may have a common substrate and function. Studying conservation of P450 genes and proteins across the *Streptomyces* genus, and examination of the genomic context in which P450s are located, can begin to reveal the importance of their role in streptomycete biology. The biochemistry of CYP105N proteins and their function within the coelibactin pathway are certainly intriguing avenues for further exploration.

## Concurrent structural resolution and biocatalytic characterization give the greatest insights

**Figure 4:**
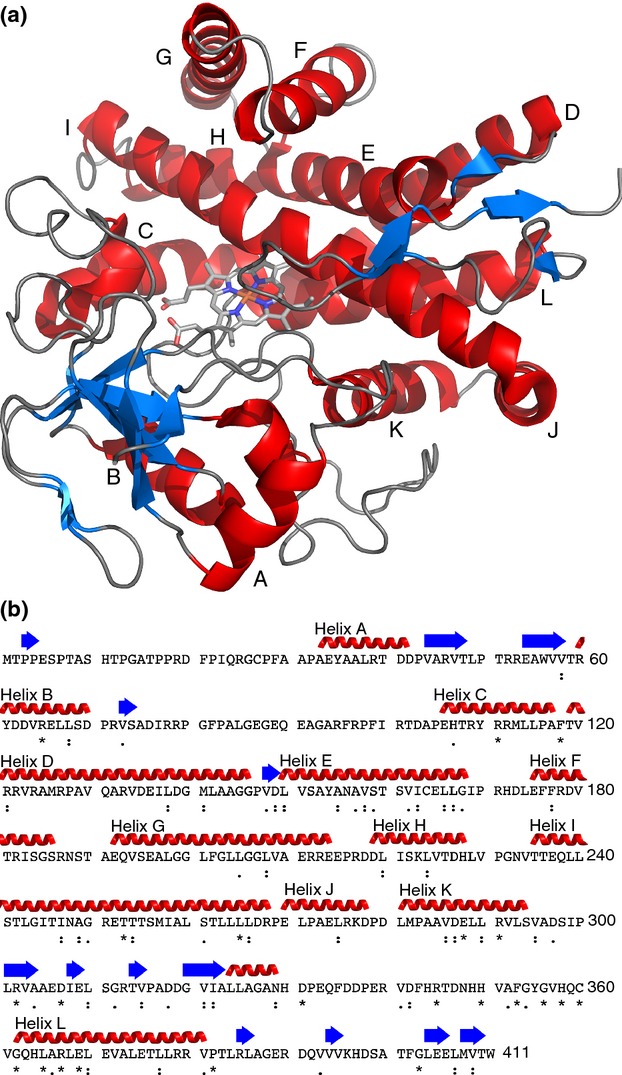
The crystal structure and sequence of CYP105N1 from *Streptomyces coelicolor*, showing important features. (a) Cartoon representation of CYP105N1 (PDB 3TYW, Zhao *et al*. 2012). Important structural features are labelled, and bound haem is shown as sticks. (b) Sequence of CYP105N1, showing secondary structural elements based on PSB 3TYW. Residues that are completely (*), strongly (:) and weakly (.) conserved (according to a ClustalW alignment) among the CYP105 family are indicated. The colour scheme used for the secondary structure elements in panel a is shown in panel b.

X-ray crystallographic determination of P450 structures has proved to be essential for elucidating the mechanisms of their biochemical function. The first bacterial P450 to have its structure resolved was P450cam (CYP101A1) in the 1980s (Poulos *et al*. 1987), and until the last decade, there was a distinct paucity of bacterial P450s in the Protein Databank. Despite the conserved nature of CYP105s across the *Streptomyces* genus, and interest in the biochemistry of this family, the first crystal structure of a CYP105, MoxA from the actinomycete species *Nonomuraea recticatena*, was not published until 2007 (Yasutake *et al*.). Several actinomycete CYP105s have since been crystallized and undergone structural resolution by X-ray crystallography. All CYP105s share the common P450 structure, although in common with the superfamily as a whole, only a small number of residues are conserved throughout the CYP105 family (Fig.4). These are mostly in haem-binding regions and common P450 motifs, such as the ExxR motif on Helix K and the FxxGx(R/H)xCxxG motif (H in CYP105) on the haem-binding loop before Helix L.

MoxA was found to be similar in function to mammalian liver P450 enzymes in having broad substrate specificity and being able to metabolize a wide variety of pharmaceutical compounds. Acceptable substrates for MoxA range from testosterone and oleanolic acid to various luciferin derivatives of human liver P450 substrates (Yasutake *et al*. 2007). Its reported catalytic abilities include demethylation of luciferin 6′-methyl ether and debenzylation (the first CYP105 reported to have this function) of luciferin 6′-benzyl ether (Yasutake *et al*. 2007). While the activity of MoxA has been biochemically characterized *in vitro,* its natural substrate and actual biological function remain unknown. It has been suggested that binding pockets in the CYP105 family are able to accommodate a wide diversity of structurally distinct substrates (Kabumoto *et al*. 2009), yet the size and shape of the binding pocket has been shown to be poorly correlated to function and is not considered predictive of substrate size (Yasutake *et al*. 2007). Thus, the broad substrate specificity demonstrated for MoxA *in vitro*, combined with the size and shape of the ligand-free binding pocket, may not supply many clues to the natural substrate. Interestingly, the crystal structures of CYP105s with broad substrate specificity do not demonstrate any obvious active site differences to those of CYP105 members with highly defined substrate specificity (Podust and Sherman 2012). The great variation seen in CYP105 substrates and products also makes prediction of putative substrates or reactions for individual CYP105s problematic. This is in contrast to some other streptomycete P450s, such as CYP170. CYP170A1 from *S. coelicolor*, the first in the family to be biochemically characterized, is known to catalyse the allylic transformation of the sesquiterpene epi-isozizaene to an epimeric mixture of albaflavenols and finally to albaflavenone (Zhao *et al*. 2008). The same catalytic ability was predicted in a variety of streptomycete species encoding proteins from different subfamilies of CYP170 (based on amino acid sequence analysis), and they were experimentally demonstrated to use the same substrate and give rise to the same product (Moody *et al*. 2012).

**Figure 5:**
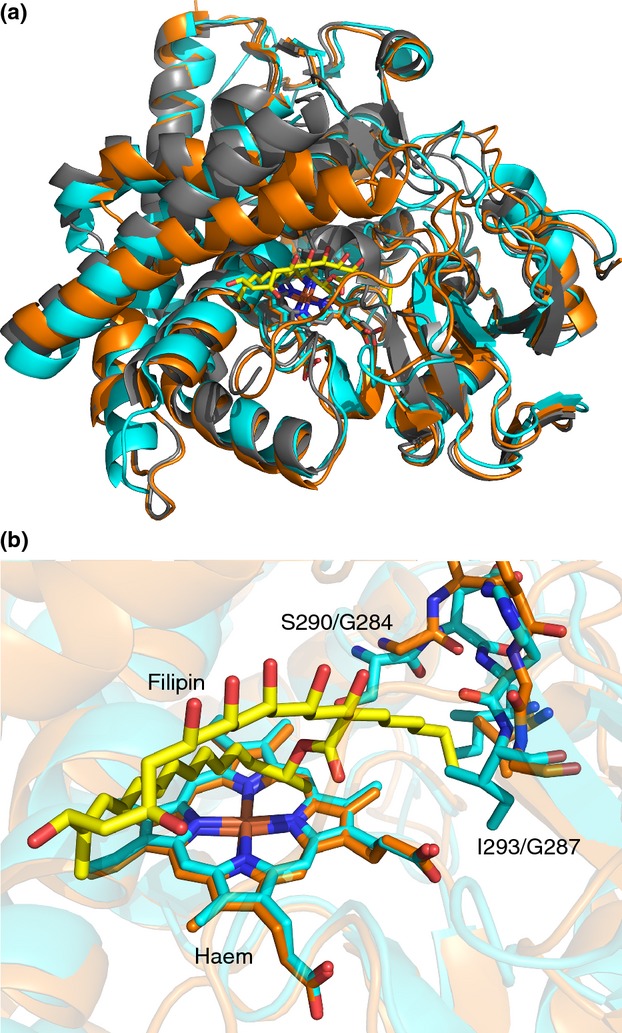
Crystal structures of CYP105D6 and CYP105P1 from *Streptomyces avermitilis*. (a) Overlaid cartoon representations of CYP105D6 ((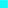) D6, PDB 3ABB; Xu *et al*. 2010), CYP105P1 ((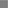) P1, PDB 3E5J; Xu *et al*. 2009) and CYP105P1 with bound filipin I ((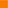) P1 +  filipin, PDB 3ABA; Xu *et al*. 2010). Haem and filipin I (from PDB 3ABA) shown as sticks. (b) Active site regions of CYP105D6 and CYP105P1, with residues responsible for the differing substrate specificity of the two enzymes (see text), bound haem and filipin I shown as sticks.

Perhaps the best structurally characterized streptomycete CYP105s are CYP105D6 and CYP105P1, both from the filipin biosynthetic pathway in *S. avermitilis*. Both proteins have been crystallized and had their structures resolved by X-ray crystallography (Xu *et al*. 2009, 2010), but importantly, CYP105P1 was also crystallized with the substrate bound (Xu *et al*. 2010) (Fig.5). In common with many P450s, the FG helices in CYP105P1 are mobile, changing from an open conformation to a closed form on ligand binding. Additionally, the BC loop, which contains 33 amino acid residues, does not have the helical structure seen in most P450s and is thought to be highly flexible (Xu *et al*. 2009). Both regions may provide access for the filipin I molecule to the haem-binding site. The crystal structures also suggested that the differing regiospecificity of the two enzymes was due to the presence of a subpocket lined with three glycine residues in CYP105P1, which was occupied by the alkyl side chain of filipin I, allowing accommodation of the bulky substrate in a specific orientation. This pocket is absent in the CYP105D6 crystal structure, with serine 290 and isoleucine 293 preventing the alkyl chain, and therefore filipin I itself, from binding in the same orientation as in CYP105P1 (Xu *et al*. 2010). This demonstrates the importance of both substrate-bound and substrate-free crystallography for elucidating the mechanisms of CYP105 catalysis and suggesting reasons for the observed stereochemistry of the product. The large difference in active site volume between the substrate-free and substrate bound structures of CYP105P1 also cautions against drawing too many conclusions from structures of a free P450 (Xu *et al*. 2009, 2010). The work conducted on CYP105D6 and CYP105P1 has allowed further appreciation of the flexibility of the binding pocket and which domains are likely to change conformation to accommodate various substrates.

**Figure 6:**
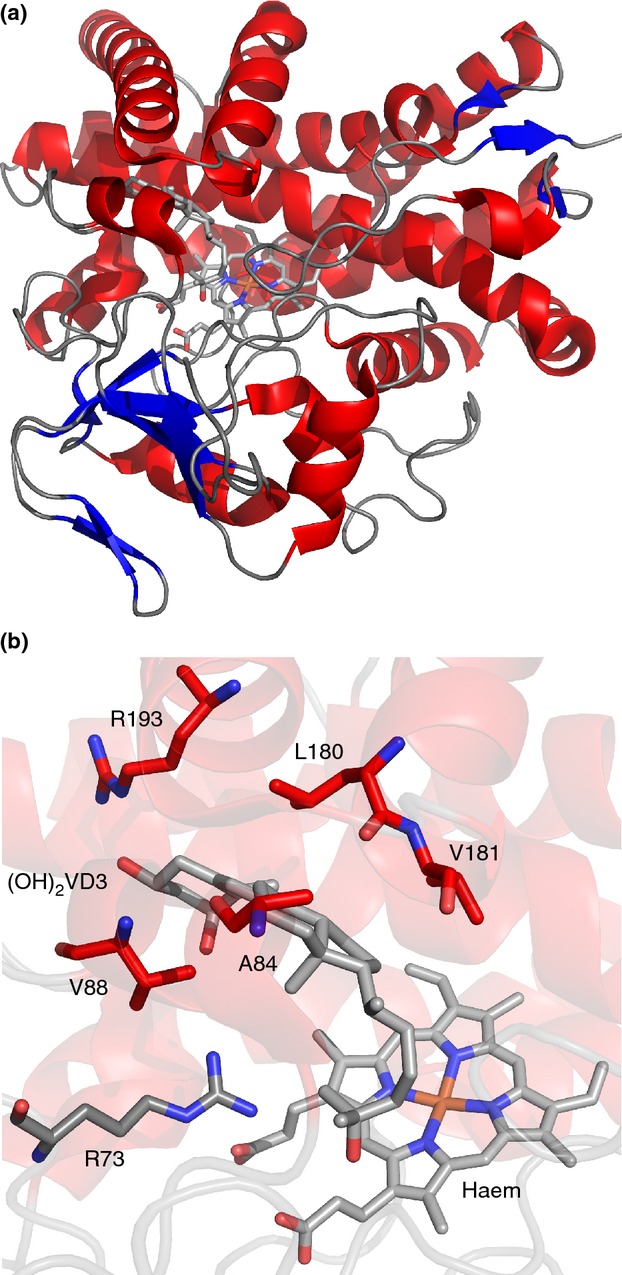
Crystal structure of CYP105A1 from *Streptomyces griseolus*. (a) Cartoon representation of CYP105A1-R84A (PDB 2ZBZ, Sugimoto *et al*. 2008) with bound haem and 1*α*,25-dihydroxyvitamin D3 ((OH)_2_D3) shown as sticks. (b) Active site region of CYP105A1-R84A, with important residues (see text), bound haem and (OH)_2_D3 shown as sticks. The same colour scheme is used for the secondary structure elements as in Fig.4.

CYP105A1 from *S. griseolus* is another interesting example. Among its other functions, this enzyme is able to catalyse the biotransformation of vitamin D3 (VD3) to 1*α*,25-dihydroxyvitamin D3 (1*α*,25-(OH)_2_D3) in two sequential hydroxylation reactions (Sawada *et al*. 2004). In mammals, VD3 is transformed from a prohormone to its active state by two separate reactions, undergoing C25 hydroxylation in the liver and 1*α*-hydroxylation in the kidney (Takeyama *et al*. 1997; Cheng *et al*. 2004). Analysis of the resolved crystal structure of CYP105A1 (Fig.6) with and without 1*α*,25-(OH)_2_D3 in the binding pocket revealed a common binding mode with human CYP2R1 for C25 hydroxylation (Sugimoto *et al*. 2008). Site-directed mutagenesis of the hydrophobic residues thought to interact with 1*α*,25-(OH)_2_D3 (V88A, L180A and V181A) all resulted in dramatic reduction in activity for both conversion of VD3 to 25-(OH)D3 and of 25-(OH)D3 to 1*α*,25-(OH)_2_D3 (Sugimoto *et al*. 2008), while R73A and R84A mutations greatly increased production of both derivatives. This suggests that the same residues are involved in controlling the specificity of both hydroxylation reactions in CYP105A1. Interaction between arginine 193 and the hydroxyl group on the substrate was also found to be important for both hydroxylation reactions, as substitutions with alanine, glutamine or lysine all decreased activity. Sugimoto *et al*. (2008) suggest that the substrate enters the binding site for the first hydroxylation event at C1, then exits and re-enters the binding pocket in a different position to allow access to C25. Clearly, given the low amino acid homology between CYP105A1 and CYP2R1 (Fig.1), prediction of the similarity in biochemistry could not have been expected based on primary sequence. This again highlights the need for both structural and biochemical characterization.

These examples demonstrate the need for structural analysis both with the substrate *in situ* and with an unoccupied binding pocket for clear understanding of the nature of the reactions catalysed by these enzymes. This is particularly apparent for CYP105N1 from *S. coelicolor*, involved in the cryptic coelibactin biosynthesis pathway, whose crystal structure (Fig.4) has been determined (Zhao *et al*. 2012) but whose biochemistry remains unknown. The predicted structure of coelibactin (Bentley *et al*. 2002) was thought structurally unlikely to bind to zinc, and it was proposed that CYP105N1 may modify the post-NRPS molecule to enable zinc chelation (Zhao *et al*. 2012). However, definitive answers are elusive without experimental evidence because of the high diversity of catalytic reactions conducted by CYP105 enzymes and the poor correlation between the observed size and shape of the binding pocket and the substrate it binds to.

Homology modelling has also been used to investigate CYP105s structurally. For example, a homology model of CYP105P2 from *Streptomyces peucetius* has been produced and docked with a flavone substrate (Kanth *et al*. 2010). It was subsequently shown that CYP105P2 is capable of oxidizing flavones *in vitro*, in almost the same position as predicted by the model (Niraula *et al*. 2012). Similarly, CYP105F2 from *S. peucetius* has been shown to catalyse specific C4 hydroxylation of the macrolide oleandomycin, and a homology model has been produced, although docking with oleandomycin was not attempted (Shrestha *et al*. 2008). It was concluded that CYP105F2 is likely to have relaxed substrate specificity, based on comparison of the homology model to the crystal structures of other P450s (Shrestha *et al*. 2008), although it has been shown elsewhere that simple comparison of binding pockets in the absence of bound substrates is uninformative (Podust and Sherman 2012). Nevertheless, these studies show that homology modelling and biochemical characterization can be combined to provide insight into CYP105 function. Despite CYP105P2 being a very close homologue of the structurally characterized CYP105P1 (Fig.1), and CYP105F2 being a close homologue of CYP105F1 from *S. lavendulae*, which is part of the biosynthesis cluster of an unknown polyketide (Mao *et al*. 1999), the native function of both enzymes remains unknown. Establishing their roles would be interesting as *S. peucetius* is not known to make macrolide secondary metabolites.

In common with other P450s, the increasing number of crystallized CYP105 proteins is a valuable resource, both for understanding the chemistry and function of individual P450s and identification of conserved traits—both physical and catalytic. While much has been learned in the past few decades from heterologous expression studies about the catalytic abilities of all P450s and specifically CYP105s, a deeper understanding of substrate binding mechanisms on the molecular level, be it through experimental determination of structures or through homology modelling and *in silico* docking, will certainly enhance intelligent design or manipulation of CYP105 systems for development and use in industrial applications. Progress in both pharmaceutical development, for example through the tailoring of molecules to reduce drug toxicity or side effects, and applications to bioremediation are more likely when the exact mechanisms of biocatalysis are fully elucidated.

## CYP105 proteins in biotechnology: utilization and manipulation

P450s have been utilized in biotechnological applications and in the production and modification of pharmaceutical compounds (e.g. Watanabe *et al*. 1995; Machida *et al*. 2009) and have proven to be amenable to manipulation in heterologous expression systems, protein purification and to rational mutagenic strategies to enhance desirable biocatalytic properties (Grogan 2011). Modifications such as alterations to the N-terminal region and adapting coding sequences to consider the codon bias of the producing cell have been tried, as have attempts at reducing secondary structures in mRNA (Zelasko *et al*. 2013). Codon bias is particularly pertinent with streptomycete P450s as the *Streptomyces* genus has an unusually high GC content (68–72%), which is not shared with a common expression host, *E. coli*. Despite the disparity in GC content and consequent coding preference, the lack of any endogenous P450s in *E. coli* makes this an ideal expression host as high levels of purity can be achieved for heterologously expressed P450s without contamination from native P450s. Bacterial P450s are, however, relatively unstable with low catalytic activity and are dependent on the presence of electron donor partners (e.g. ferredoxin and ferredoxin reductase) which makes them less suitable for bulk production and more useful in high specification production such as the pharmaceutical industry (Sakaki 2012). In their favour, the bacterial P450s thus far identified are cytosolic, as opposed to many eukaryotic P450s which are membrane bound. This is a clear advantage in terms of downstream biotechnological applications as bacterial P450s are soluble, whereas eukaryotic P450s require detergent treatment to solubilize them prior to further use (Munro *et al*. 2013). Perhaps as a consequence of this, bacterial P450s have been easier to characterize than fungal or plant P450s both in terms of function and structure (Podust and Sherman 2012), leading to more opportunities for their development as biotechnological tools.

**Figure 7:**
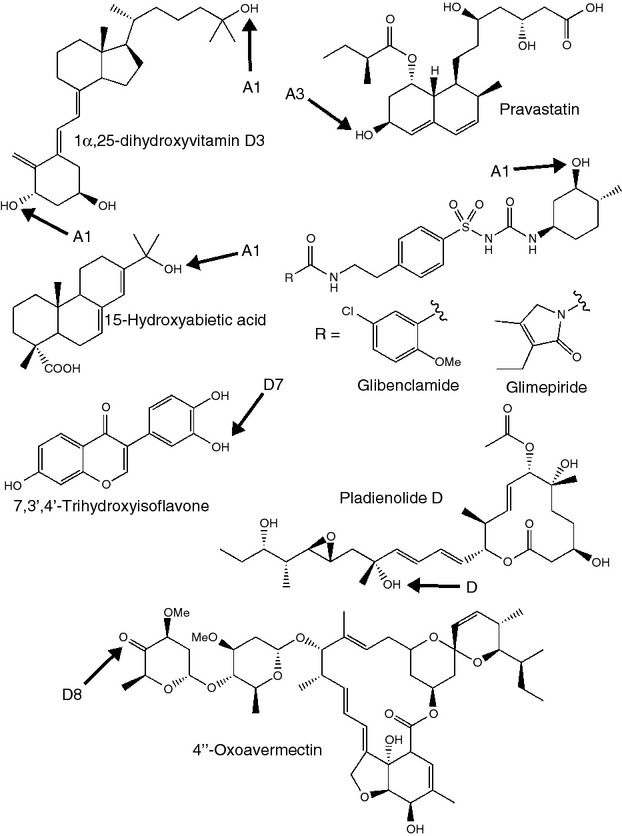
Compounds produced by biotechnological use of CYP105s. The structural feature introduced by the CYP105 is arrowed, and the CYP105 involved is indicated.

One of the most successful industrial applications of CYP105 biocatalysis is in the production of the anti-hyperlipidaemia drug, pravastatin. Compactin (mevastatin), a metabolite of the fungus *Penicillium citrinum*, undergoes biotransformation by CYP105A3 (also called P450sca-2) in *Streptomyces carbophilus* to pravastatin *via* a stereoselective 6*β*-hydroxylation (Watanabe *et al*. 1995) (Fig.7). The purified CYP105A3 has, more recently, been subject to site-directed mutagenesis. A mutant with six amino acid substitutions (G52S/F89I/P159A/D269E/T323A/E370V) gave improved biotransformative activity in whole cell extracts compared with the wild type when heterologously expressed in *E. coli*, and this was attributed to improvements in expression of active protein (Ba *et al*. 2013a). Further mutagenic studies targeted the redoxin interaction interface (T119 and N363), the substrate access channel (V194) and one residue within the binding pocket (T85), with a view to improving the enzyme kinetics. The resulting improved activity, particularly related to the altered redoxin interface residues, was attributed to enhanced electron transfer in the P450 redox system (Ba *et al*. 2013b). Similarly, CYP105A1 is used industrially to catalyse the biotransformation of VD3 to 1α,25-dihydroxyvitamin D3 (Sugimoto *et al*. 2008; see above), an important pharmaceutical product for the treatment of osteoporosis and chronic renal failure among others. Site-directed mutagenesis was again used to enhance the activity of this P450. Mutation of the two arginine residues identified in structural studies (Sugimoto *et al*. 2008; see above), to form the double mutant R73V/R84A, gave large increases in the activity of both hydroxylation events (400-fold for 25-hydroxylation and 100-fold for 1*α*-hydroxylation) (Hayashi *et al*. 2008). This progress may herald a new era in P450 utilization, with enzymes used in well-established processes being intelligently manipulated to increase their usefulness.

One filipin-producing species, *Streptomyces miharaensis*, has been proposed as a biocontrol agent against a range of commercially important fungal plant pathogens. The filipin complex is a mixture of polyene macrolides hydroxylated in slightly different positions, requiring the action of a CYP105D and a CYP105P. This mixture is produced by a variety of streptomycete species and displays potent antifungal activity (Ammann and Gottlieb 1955; Whitfield *et al*. 1955). Kim *et al*. (2012) reported *S. miharaensis*-mediated inhibition of *Alternaria mali*, *Aspergillus niger* and the causative agent of Fusarium wilt, *Fusarium oxysporum* f.sp. lycopersici, commensurate with the activity of commercially available fungicide ‘Benomyl’ (without the toxicity to invertebrates that this synthetic antifungal exhibits). The filipin biosynthetic pathway, including the P450s, may be amenable to genetic engineering to alter the structure and fungicidal specificity of the products. This has been achieved in *Streptomyces* previously for polyene macrolide biosynthetic pathways which include P450 enzymes. For example, nystatin derivatives were generated by disruption of one of the P450 enzymes involved in oxidative tailoring modifications during the final steps of nystatin (Fig.3) biosynthesis (Brautaset *et al*. 2011). The possibility, therefore, of developing a range of fungicidal compounds based on filipin biosynthesis in streptomycetes is an attractive one.

Heterologous expression of the CYP105A1 from *S. griseolus* in *Bacillus megaterium* MS941 allowed high scale production of 3-hydroxylated derivatives of the sulfonylurea drugs glibenclamide and glimepiride (Kleser *et al*. 2012) (Fig.7). These are widely used in the treatment of type 2 diabetes, which is increasingly common as it is often a consequence of being overweight or obese and new drugs for effective treatment are constantly sought (Banerji and Dunn 2013). Both glibenclamide and glimepiride are known to increase insulin secretion and promote normoglycaemia, thereby mitigating the extensive systemic damage that can be caused by the hyperglycaemia associated with diabetes. Hydroxylated derivatives of the sulfonylurea drugs could be used as platform compounds for development of the next generation of diabetic medicines. Interestingly, two expression systems were tried, with and without a complementary redox system also being expressed. No evidence was reported of an increase in productivity with the additional redox system, suggesting *B. megaterium* is able to provide the necessary electron transfer system (Kleser *et al*. 2012). The extremely versatile CYP105A1 has also been expressed in *E. coli* with redox partners from *Schizosaccharomyces pombe* and used to catalyse the hydroxylation of the resin compound abietic acid at C15 to give 15-hydroxyabietic acid (Janocha *et al*. 2013) (Fig.7). This compound can then be used to synthesize 15-hydroperoxyabietic acid (15-HPA), a major allergen. Production of 15-HPA in pure form using bioconversion by CYP105A1 promises to be useful in allergy testing (Janocha and Bernhardt 2013).

Other potential biotechnological uses for CYP105s include the conversion of diosgenin to isonuatigenone by CYP105C from *S. virginiae* IBL14, described above (Fig.2) (Wang *et al*. 2007, 2009b). CYP105D7, involved in pentalenic acid biosynthesis in *S. avermitilis*, has also been shown to catalyse regiospecific hydroxylation of isoflavones such as daidzein both *in vitro* and *in vivo* (Pandey *et al*. 2010). The product of daidzein oxidation, 7,3′,4′-trihydroxyisoflavone (Fig.7), is of interest due to its enhanced antioxidant, anti-inflammatory and anti-allergenic activities compared to daidzein itself (Park *et al*. 2008). CYP105D8, CYP105B2, CYP105AA1 and CYP105AA2 from strains of *Streptomyces tubercidicus* were all found to catalyse oxidation of the 4″-hydroxy group of avermectin to form 4″-oxoavermectin (Jungmann *et al*. 2005) (Fig.7). Biotechnological production of 4″-oxoavermectin would reduce the synthetic route to from avermectin to emamectin (4″-deoxy-4″-methylaminoavermectin B), an agriculturally important insecticide, from four steps (Cvetovich *et al*. 1994) to two, by eliminating the need for protection of other alcohol functionalities during oxidation to 4″-oxoavermectin. Indeed, if *S. avermitilis* could be engineered to produce 4″-oxoavermectin directly, only a single step would then be required for conversion to emamectin.

An excellent example of a CYP105 enzyme being utilized in a biotechnological application is seen in PsmA, a CYP105D homologue from *Streptomyces bungoensis* A-1544, for the production of the macrolide pladienolide D (Fig.7). Pladienolides are 12 membered ring structures produced as specialized metabolites by *Streptomyces platensis* Mer-11107, by the action of a PKS and several post-PKS modifying enzymes (including a P450 belonging to the CYP107 family) (Machida *et al*. 2008b). Pladienolides are of interest because they show potent anti-tumour activity, targeting the protein component of splicing factor SF3b. Pladienolide D, which is a minor product, was reported as the most promising therapeutic candidate, and a derivative of this compound was found to be the most potent and have the best physicochemical properties for further pharmaceutical development (Machida *et al*. 2008a). *Streptomyces bungoensis* A-1544 was found to catalyse the hydroxylation of pladienolide B, the major pladienolide, to pladienolide D. Overexpression of *psmA* resulted in a 15-fold increase in biotransformation of pladienolide B to pladienolide D (Machida *et al*. 2008a). A further development saw the heterologous expression of PsmA in *S. platensis*, allowing high level pladienolide D production in a one-step process (Machida *et al*. 2009). A semi-synthetic analogue, E7107, has shown such potency for inducing remission in lung cancer models, it has progressed to clinical trials (Podust and Sherman 2012).

## Future perspectives

CYP105s form a fascinating family of enzymes. Historically investigated for their roles in xenobiotic degradation, they are more recently revealed as essential components in several diverse biosynthetic clusters and appreciated for their versatility in biotechnological applications. Further work will no doubt extend the industrial roles to which these enzymes can be applied, and the clinical uses that the bioactive natural products they help biosynthesize can be put to, utilizing the ability of CYP105s to accommodate a wide variety of substrates to generate structurally diverse or novel compounds for further use. In ecological terms, there remains much we do not know about CYP105s. The high level of conservation of this family across the genus argues for important functions within the cell, but without investigation of the enzymes *in vivo* with consideration of their natural environment, their intrinsic value to the cell remains a mystery.
